# CONCEPTUALIZING PERSON-CENTEREDNESS

**DOI:** 10.1093/geroni/igae098.0676

**Published:** 2024-12-31

**Authors:** Lea Efird-Green, Sheryl Zimmerman, Lauren Stratton, Philip Sloane, Sam Fazio

**Affiliations:** University of North Carolina at Chapel Hill, Chapel Hill, North Carolina, United States; University of North Carolina at Chapel Hill (UNC), Chapel Hill, North Carolina, United States; Alzheimer’s Association, Chicago, Illinois, United States; University of North Carolina, Chapel Hill, North Carolina, United States; Alzheimer’s Association, Chicago, Illinois, United States

## Abstract

The concept of person-centeredness (PC) has become commonplace in health and supportive care, and perhaps for this very reason, there is no agreed upon understanding as to what constitutes “person-centeredness.” To clarify the concept, a systematic review of current definitions of PC in the literature was conducted; it revealed that most papers referencing person-centered or patient-centered interventions do not define the term, and that definitions that when they do, they vary widely. Given the benefit of having a common understanding of PC, think-tank meetings were held with 65 experts in the fields of health and aging policy, research, and practice. The experts were provided numerous prompts (e.g., in the context of being person-centered, how do issues of dependency and competency enter in?); responses indicated that they conceptualize and implement the concept of PC differently at the macro, meso, and micro levels. At the macro level, there is a focus on core values, the influence of privilege and oppression, and how PC is implemented – or not – in systems and cultures. On the meso level, there is a focus on organizational responsibility, the applicability of PC to various communities, and the importance of relationships. On the micro level, PC is focused on care, including individualization and personalization, the importance of care partners/family, and the limitations of PC in individual settings. This presentation will address the three levels of PC, including each level’s focus, implementation, and challenges, with the goal of establishing a multilevel conceptualization of PC.

